# Supplementary material for: Surface-kinetics mediated mesoporous multipods for enhanced bacterial adhesion and inhibition
Source: Nat Commun. 2019 Sep 26;10:4387. doi: 10.1038/s41467-019-12378-0 (PMC6763480; doi:10.1038/s41467-019-12378-0)
Supplement: Supplementary file 1 — Supplementary Information [file 41467_2019_12378_MOESM1_ESM.pdf]

Supplementary Information

Surface-Kinetics Mediated Mesoporous Multipods for Enhanced Bacterial Adhesion and Inhibition

Zhao *et al.*

Supplementary Figures

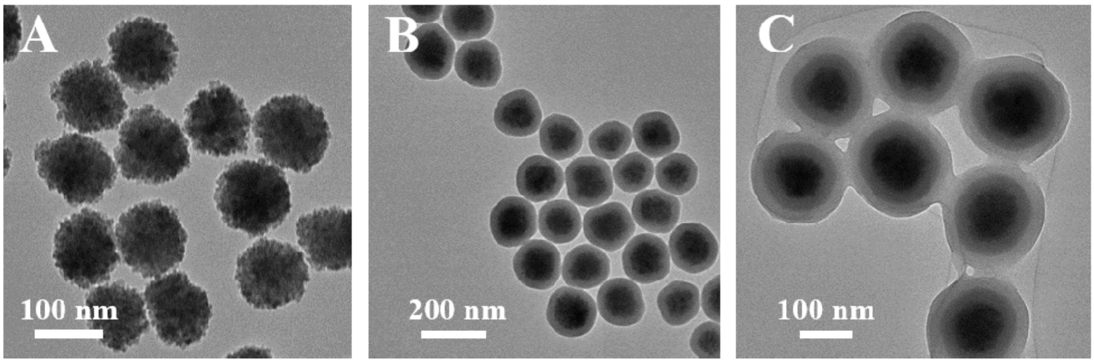

Supplementary Figure 1. Characterization on morphologies of the obtained nanoparticles. The TEM images of (A) magnetic Fe_3O_4, (B) core-shell Fe_3O_4@SiO_2 and (C) double shell Fe_3O_4@SiO_2@RF nanoparticles. The Fe_3O_4 nanoparticles are prepared through solvothermal method[1], and successive coating of SiO_2 and RF are achieved through modified Stöber methods[2,3]. It can be seen that the magnetic Fe_3O_4 nanoparticles have a diameter of 100 nm, the Fe_3O_4@SiO_2 nanoparticles have a diameter of 180 nm and the Fe_3O_4@SiO_2@RF nanoparticles have a diameter of 260 nm. All particles are well dispersed and uniform in size.

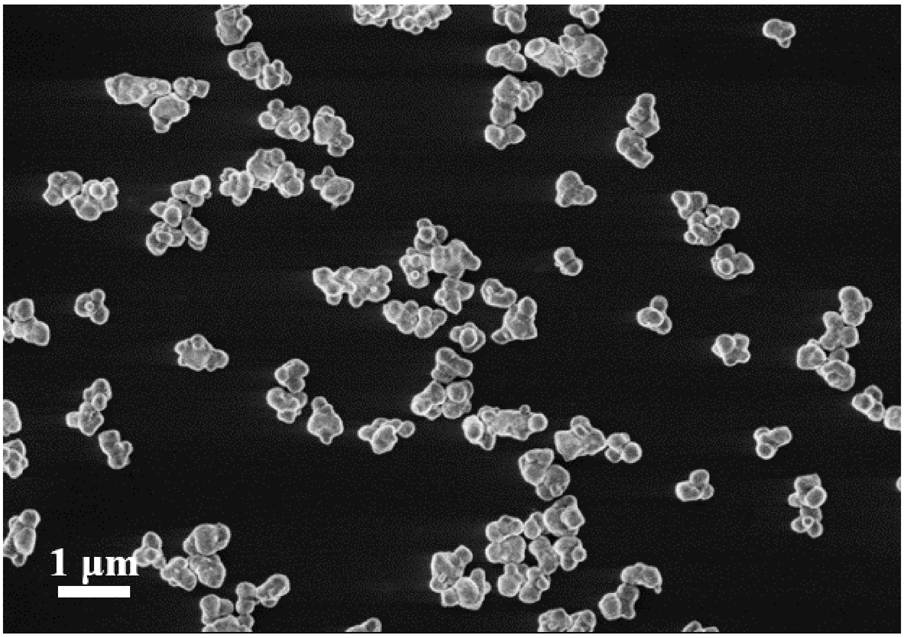

Supplementary Figure 2. Large area SEM image of the tetra-pods nanoparticles. The SEM image of the tetra-pods Fe_3O_4@SiO_2@RF&PMOs nanoparticles prepared through the surface-kinetics mediated multi-site nucleation of PMOs on Fe_3O_4@SiO_2@RF, showing that all the particles are uniform and well dispersed, the tribulus-like tetra-pods structure can be clearly observed.

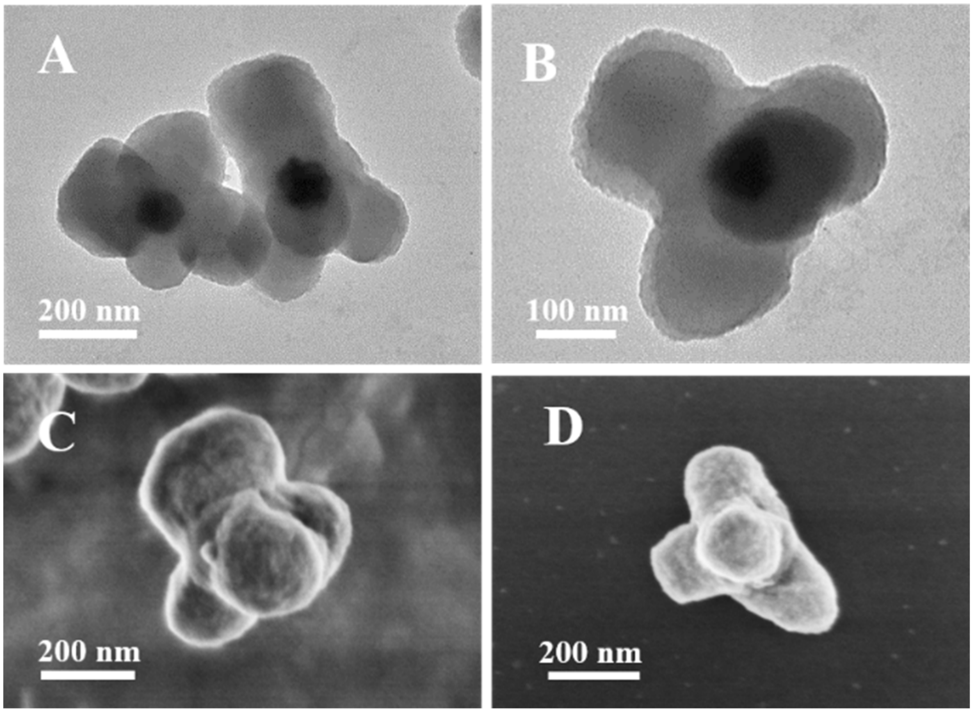

Supplementary Figure 3. Characterization on morphology of the tetra-pods nanoparticles. (A, B) TEM images and (C, D) SEM images of the tetra-pods Fe_3O_4@SiO_2@RF&PMOs nanoparticles recorded from different angles, clearly showing four PMO cubes located on one nanosphere core. The structure is similar to a tetrahedral symmetry.

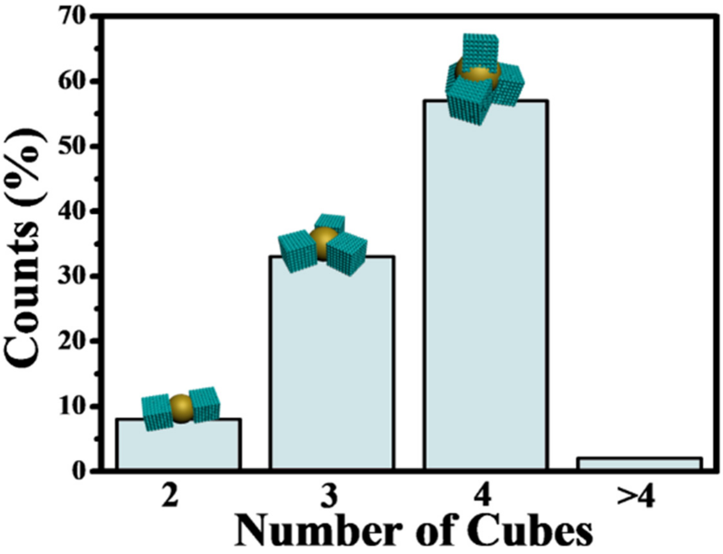

Supplementary Figure 4. Diversity of morphologies in the tetra-pods structured sample. The statistic on number of the PMO cubes on each Fe_3O_4@SiO_2@RF&PMOs nanoparticle (tetra-pods structure) based on the observations of 100 randomly selected nanoparticles from TEM and SEM images. The result indicates that most of the multipods nanocomposites possess symmetric tetra-pods topologic structure. Source data are provided as a Source Data file.

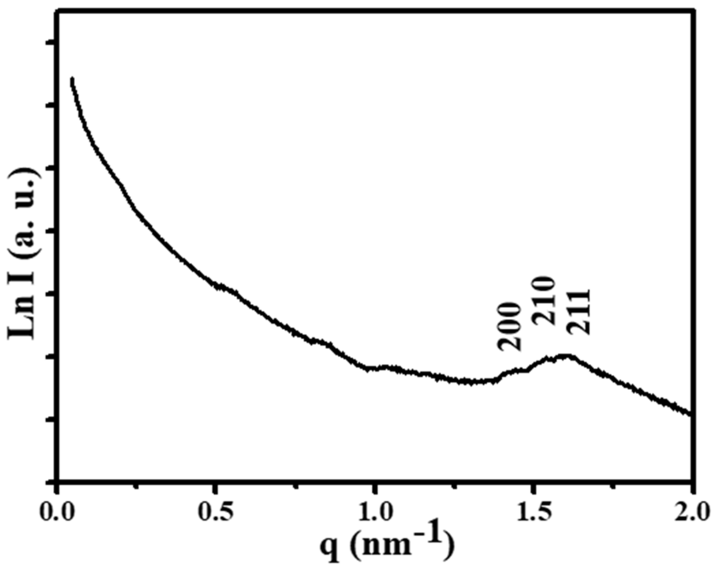

Supplementary Figure 5. Characterization on mesostructure of tetra-pods nanocomposites. The small-angle X-ray scattering (SAXS) patterns of the mesoporous tetra-pods Fe_3O_4@SiO_2@RF&PMOs nanoparticles prepared by the surface-kinetics mediated multi-site nucleation method. Three scattering peaks are appeared at 1.35, 1.51, and 1.65 nm^{-1}, corresponding to the 200, 210, and 211 reflections of space group $Pm\bar{3}n$ mesostructure, indicating that the PMO pods domains are cubic mesostructure.

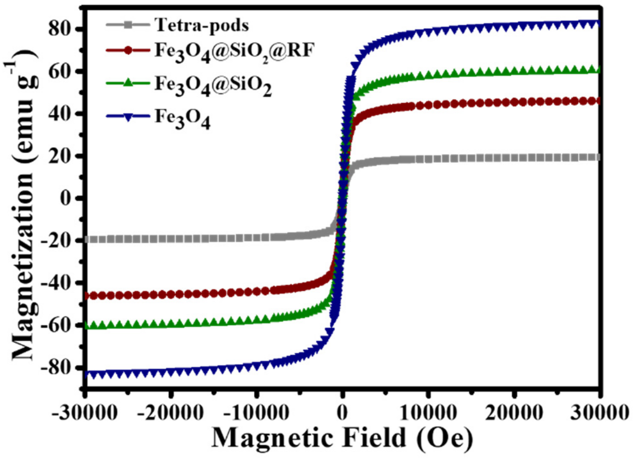

Supplementary Figure 6. Characterizations on magnetic property of the nanoparticles. The magnetization curves of the Fe_3O_4 nanoparticles, Fe_3O_4@SiO_2 nanoparticles, Fe_3O_4@SiO_2@RF nanoparticles and tetra-pods structured Fe_3O_4@SiO_2@RF&PMO nanoparticles. It can be seen that the obtained tetra-pods structured Fe_3O_4@SiO_2@RF&PMO nanoparticles exhibit superparamagnetic properties with a high saturation magnetization value of ~ 20 emu g^{-1}, enabling the nanoparticles for magnetic induced separation. The saturation magnetization value of the nanoparticles is decreased after SiO_2, RF coating and PMO cubes growth due to increase of none-magnetic components.

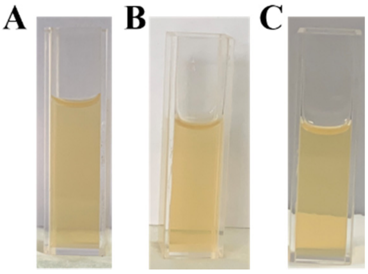

Supplementary Figure 7. Stability test of the nanoparticles in Luria-Bertani (LB) solution. Digital photos of LB solutions of the tetra-pods structured Fe_3O_4@SiO_2@RF&PMOs nanoparticles after different periods of times: (A) 0 h, (B) 6 h and (C) 12 h. The solutions maintain clear with none observable suspended matter, indicating the good stability of the multipods nanoparticles in LB solution.

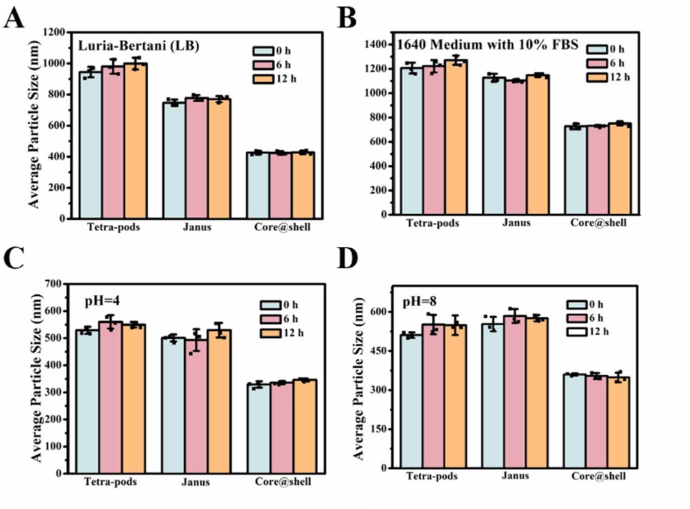

Supplementary Figure 8. Stability test of the nanoparticles in various solutions. Average particle diameter acquired from dynamic light scattering (DLS) measurements of the tetra-pods structured Fe_3O_4@SiO_2@RF&PMOs nanoparticles, Janus structured Fe_3O_4@SiO_2@RF-PVP&PMO nanoparticles and core@shell structured Fe_3O_4@SiO_2@RF@PMO nanoparticles in a series of different solutions: (A) Luria-Bertani (LB), (B) Roswell Park Memorial Institute1640 medium with 10 % fetal bovine serum (FBS), (C) pH = 4 and (D) pH = 8 solutions for different periods of times. These results show that the diameters of the nanoparticles remain nearly constant in different solutions, indicating no aggregations of the nanoparticles in these solutions. The bars represent mean ± s.d. derived from n = 3 groups of nanoparticle suspension. Source data are provided as a Source Data file.

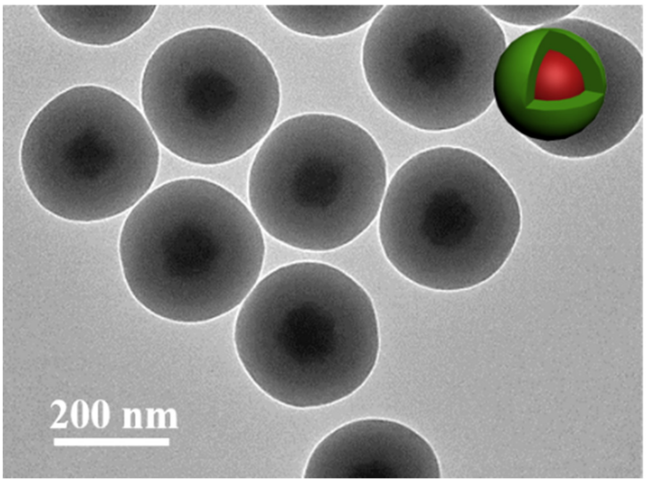

Supplementary Figure 9. Characterization on morphology of the Fe_3O_4@SiO_2 nanoparticles. The TEM image of the core@shell Fe_3O_4@SiO_2 nanoparticles with a diameter of 260 nm prepared by a modified Stöber method[2]. Inset is the 3D structural model of the core@shell Fe_3O_4@SiO_2 nanoparticle.

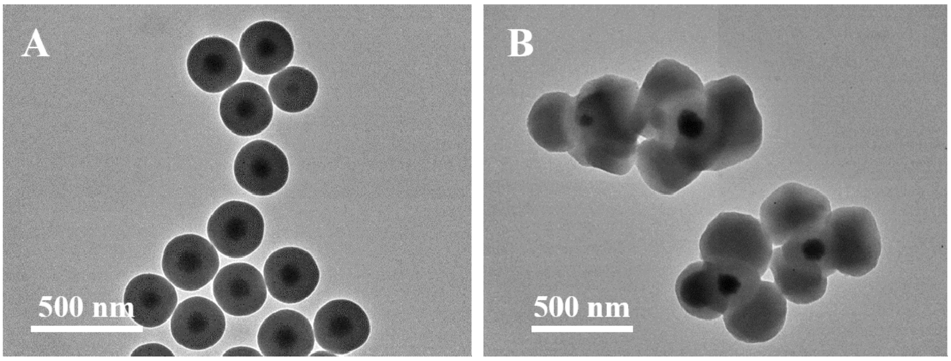

Supplementary Figure 10. Characterization on morphologies of different nanocomposites. TEM images of the core@shell Fe_3O_4@RF nanoparticles (A) with a diameter of 260 nm prepared by a modified Stöber method[3] and the multipods Fe_3O_4@RF&PMOs nanocomposites (B) prepared by the surface-kinetics mediated multi-site nucleation strategy. At the same reaction conditions, the obtained multipods Fe_3O_4@RF&PMOs nanocomposites have identical morphology with the multipods Fe_3O_4@SiO_2@RF&PMOs nanoparticles, indicating that SiO_2 middle layer of the Fe_3O_4@SiO_2@RF does not affect the surface topological structure of the final nanocomposites after multi-site nucleation and growth of PMOs.

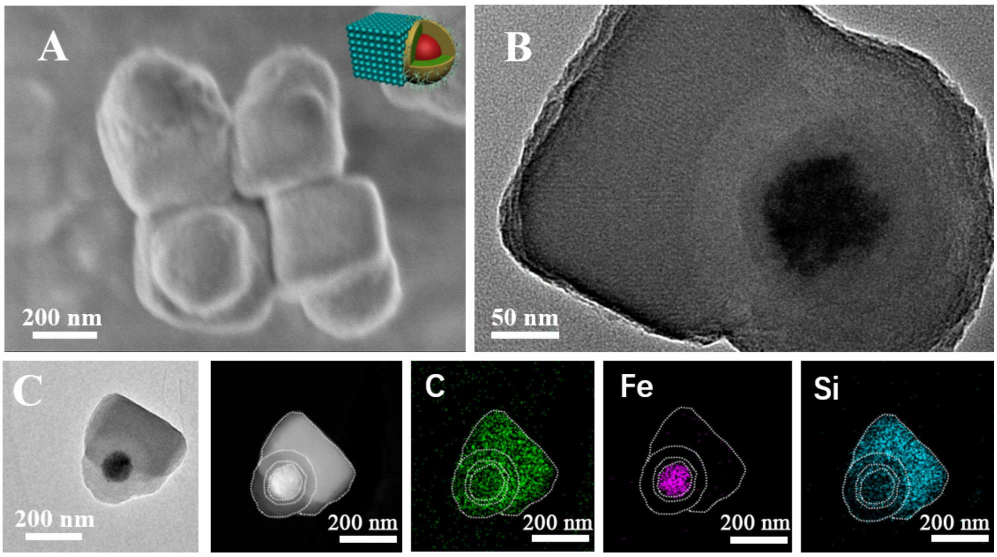

Supplementary Figure 11. The growth of PMO cubes on nanoparticles with PVP surface. (A) SEM, (B) TEM images and (C) element mapping of the Janus Fe_3O_4@SiO_2@RF-PVP&PMO nanoparticles. The particles were fabricated in the same process as that of the tetra-pods Fe_3O_4@SiO_2@RF&PMOs nanoparticles, except that the Fe_3O_4@SiO_2@RF nanoparticles were pre-modified with PVP to suppress multi-site assembly of PMOs. All the expected elements (iron & carbon in the Fe_3O_4@SiO_2@RF core and silicon in the PMO cube) were detected and matched well with the relative positions in the Janus nanocomposite. Inset (A) is the 3D structural model of Janus Fe_3O_4@SiO_2@RF-PVP&PMO nanoparticle.

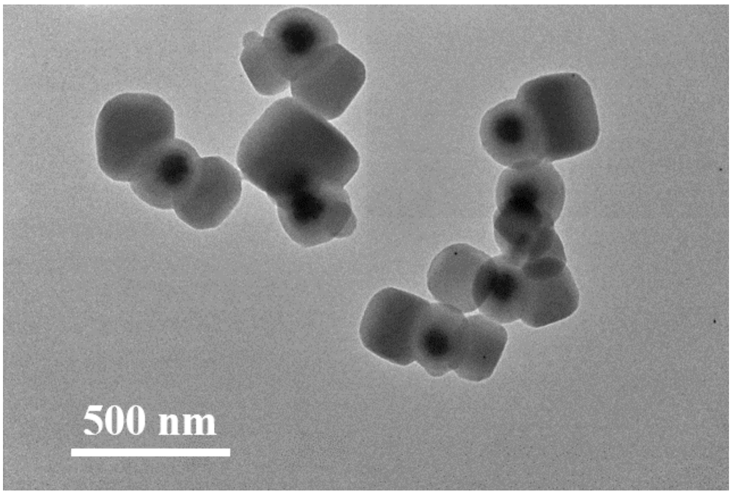

Supplementary Figure 12. The growth of PMO cubes on nanoparticles with PVP surface. The TEM image of the dual-pods Fe_3O_4@SiO_2@RF-PVP&PMO nanoparticles prepared through growing PMO cubes on PVP modified Fe_3O_4@SiO_2@RF nanoparticles. The Fe_3O_4@SiO_2@RF nanoparticles were stirred in PVP solution for 5 min, which is shorter than that for the preparation of Janus Fe_3O_4@SiO_2@RF-PVP&PMO nanoparticles (30 min, see the experimental section in supporting information for details). Only one or two PMO cubes were grown on each nanoparticle, indicating that the multi-site nucleation growth was partially suppressed by PVP coverage.

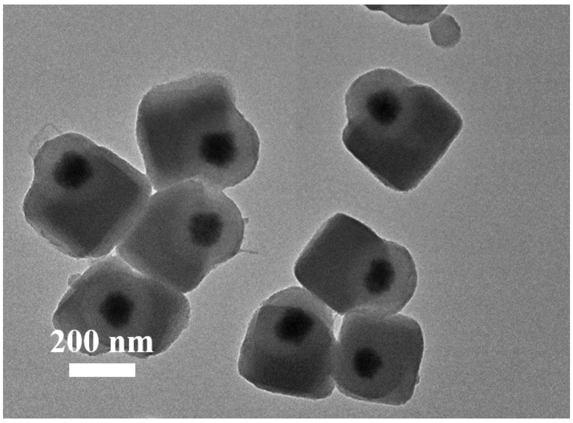

Supplementary Figure 13. The growth of PMO cubes on nanoparticles with PSS surface. The TEM image of the Janus Fe_3O_4@SiO_2@RF-PSS&PMO nanocomposites obtained from the growth of PMO on the poly(sodium-p-styrenesulfonate) (PSS) modified Fe_3O_4@SiO_2@RF nanospheres. Similar to PVP (Fig. 2C), the surface modification with PSS can also hinder the multi-site nucleation of PMOs on RF surface, resulting in Janus architectures.

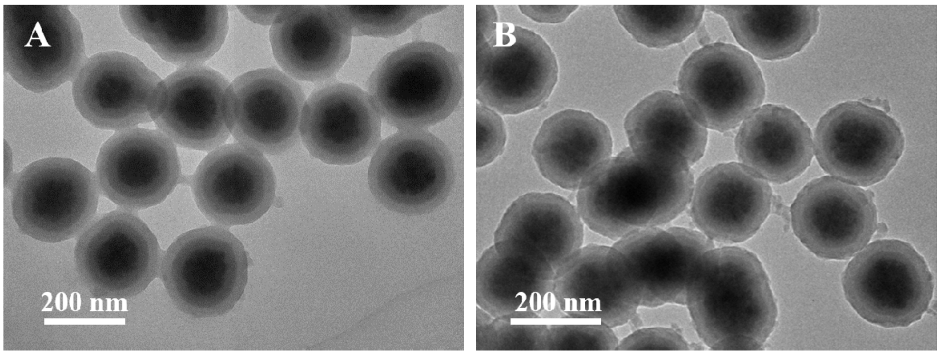

Supplementary Figure 14. The nanoparticles with different functional groups. TEM images of (A) phenolic amino group functionalized Fe_3O_4@SiO_2@RF-A nanoparticles and (B) phenolic nitro group functionalized Fe_3O_4@SiO_2@RF-N nanoparticles with the diameter of around 260 nm. The results indicating that the morphologies and sizes of the nanoparticles are well retained after the introduction of the phenolic amino and phenolic nitro groups in the framework of RF layers.

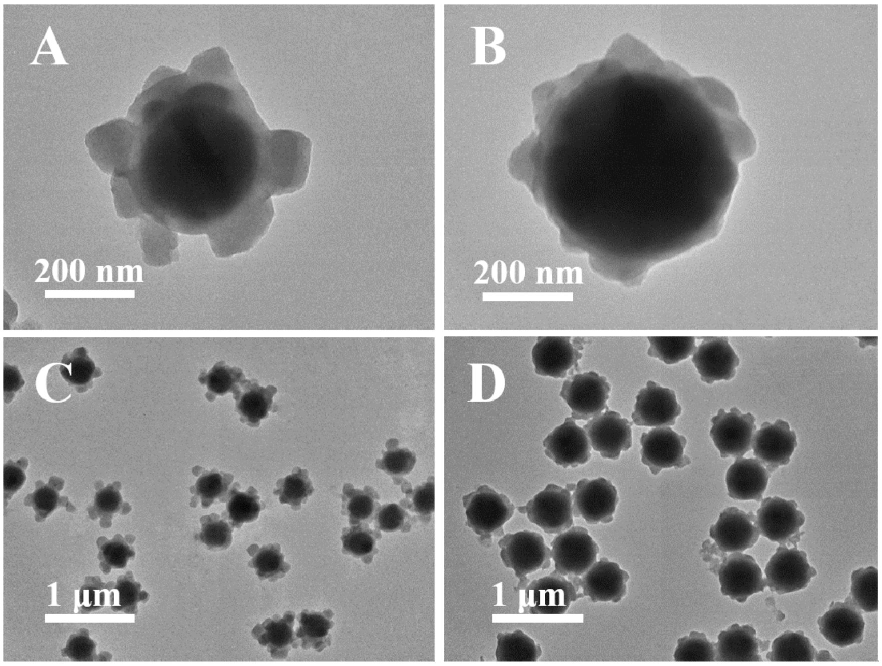

Supplementary Figure 15. The growth of PMO cubes on different sized nanoparticles. TEM images with different magnifications of the multipods SiO_2@RF&PMOs nanoparticles grown on the core@shell SiO_2@RF nanoparticles with different diameter: (A, C) 300 nm, (B, D) 400 nm. The results indicate that the number of PMO cubes on the particle surface can increase as the particle size.

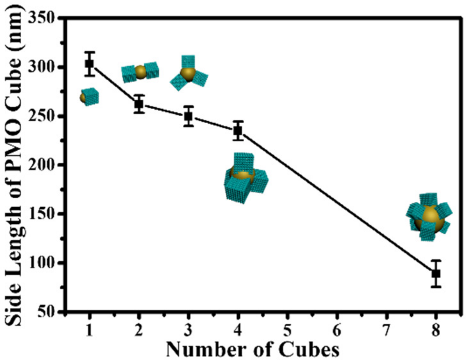

Supplementary Figure 16. The relation between the size and number of PMO cubes. The correlation between the size and number of PMO cubes on Fe_3O_4@SiO_2@RF with different diameters. The average side length of PMO cubes decrease from 300 to 100 nm as increasing of the number of the PMO cubes on the Fe_3O_4@SiO_2@RF surface from 1 to 8. The bars represent mean ± s.d. derived from n = 20 randomly selected nanocubes. Source data are provided as a Source Data file.

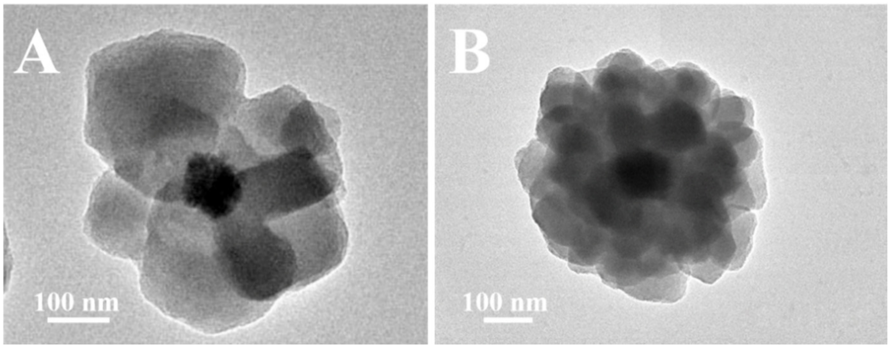

Supplementary Figure 17. The growth of PMO cubes on mesoporous RF surface. TEM images of the multipods structured Fe_3O_4@mRF&PMOs nanoparticles prepared from the multi-sites nucleation of PMOs on Fe_3O_4@mRF nanoparticles with different diameters (A, 200 nm; B, 300 nm). It can be seen that the number of nucleation sites is greatly increased as the diameter of Fe_3O_4@mRF nanoparticles. The tendency is the same as that of nanoparticles coated with nonporous RF.

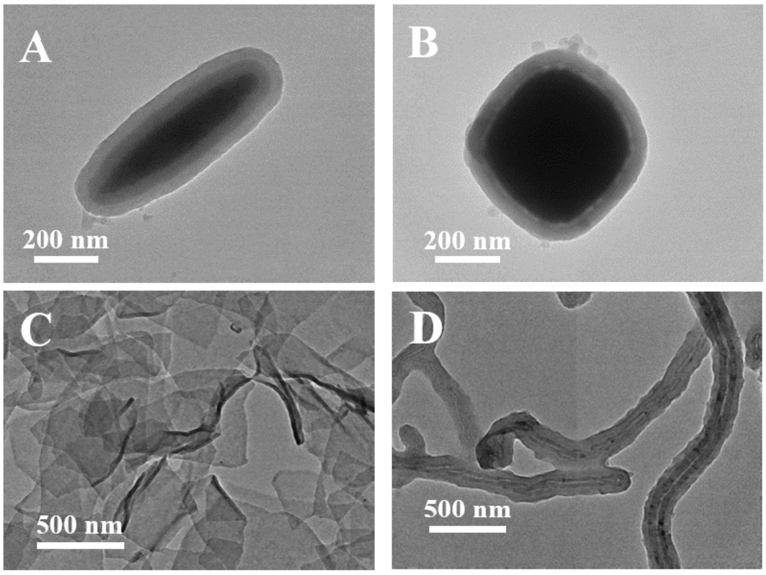

Supplementary Figure 18. Various shaped nanomaterials with RF surface. TEM images of the nanomaterials with various morphologies coated with RF layer based on a modified Stöber method[3]. (A) spindle Fe_2O_3@SiO_2@RF, (B) cubic Fe_2O_3@SiO_2@RF, (C) graphene@RF and (D) CNT@RF (CNT = carbon nanotube). It can be seen that RF layers are uniformly coated on their surfaces.

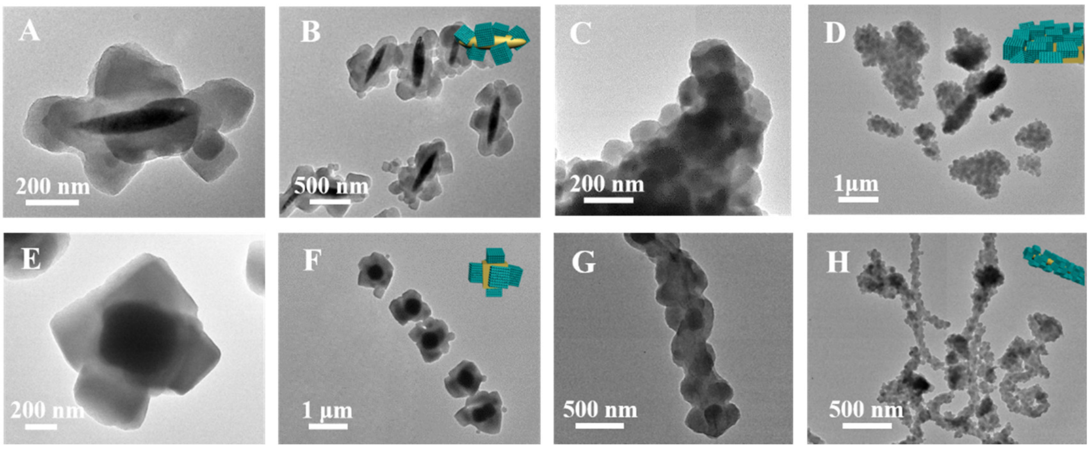

Supplementary Figure 19. The formation of multipods topology on various nanomaterials. TEM images with different magnification of the multipods structured nanomaterials with different morphologies and functions: (A, B) spindle Fe_2O_3@SiO_2@RF&PMOs, (C, D) Graphene@RF&PMOs, (E, F) cubic Fe_2O_3@SiO_2@RF&PMOs and (G, H) CNT@RF&PMOs nanocomposites. The nanomaterials were obtained through the surface-kinetic mediated growth of PMOs on different substrates. The results clearly indicate that after the surface modification of RF layers, the surface topologic structure of the nanomaterials can be further tuned by the multiple nucleation and growth of PMO pods on the RF surface.

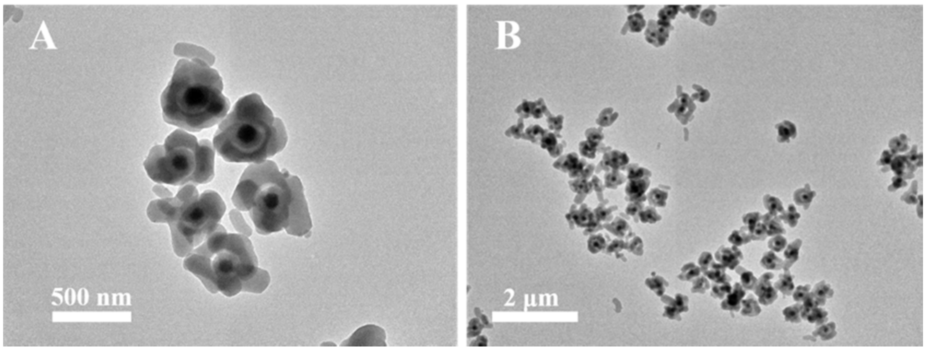

Supplementary Figure 20. The growth of multiple PMO rods on the surface of nanoparticles. TEM images with (A) high and (B) low magnifications of the multipods Fe_3O_4@SiO_2@RF&PMOs nanoparticles, which are composed of Fe_3O_4@SiO_2@RF nanosphere as a center and multiple PMO nanorods as "pods" on the RF surface. These results indicate that the surface-kinetics mediated multi-site nucleation strategy can also be utilized for multi-site nucleation and growth of PMO nanorods with hexagonal (p6mn) mesopore structure on the RF surface.

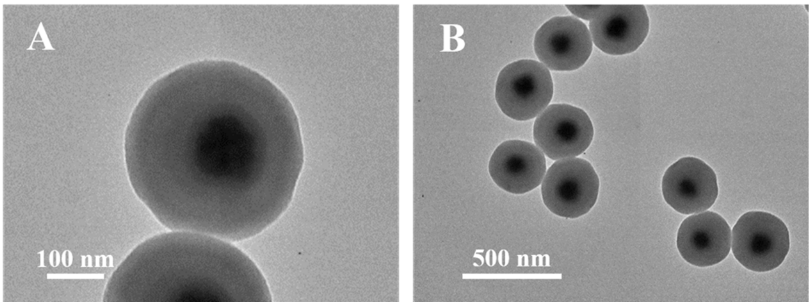

Supplementary Figure 21. The morphology of Fe_3O_4@SiO_2@RF@PMO nanoparticles. TEM images with (A) high and (B) low magnifications of the core@shell structured Fe_3O_4@SiO_2@RF@PMO nanoparticles. It can be seen that the obtained nanoparticles are uniform spherical shape with diameter of ~ 320 nm.

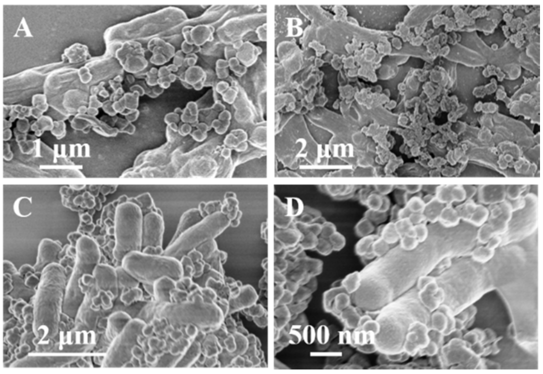

Supplementary Figure 22. The adherence of tetra-pods nanoparticles on *E. coli*. The typical SEM images with (A-C) low and (D) high magnifications of the tetra-pods Fe_3O_4@SiO_2@RF&PMOs nanoparticles adhered on *E. coli* surfaces. The images do not show any significant variations.

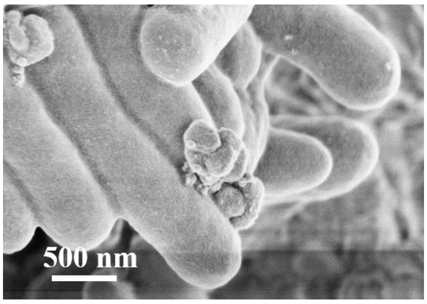

Supplementary Figure 23. The adherence of Janus nanoparticles on *E. coli*. SEM images of the Janus Fe_3O_4@SiO_2@RF&PMO nanoparticles adhered on *E. coli* surfaces, showing that the amount of adhesive nanoparticles is very few. It clearly indicates that the Janus nanoparticle cannot efficiently adhere on bacteria.

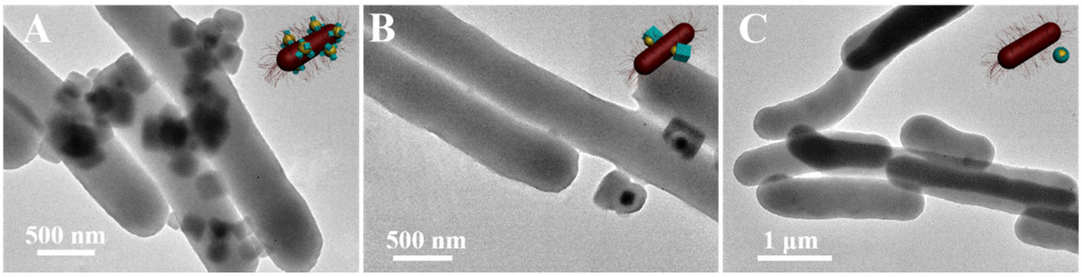

Supplementary Figure 24. The adherence of various nanoparticles on *E. coli*. TEM images of (A) tetra-pods Fe_3O_4@SiO_2@RF&PMOs (T-RF&PMOs) nanoparticles (B) Janus Fe_3O_4@SiO_2@RF&PMO (J-RF&PMO) and (C) core@shell Fe_3O_4@SiO_2@RF@PMO (RF@PMO) nanoparticles adhered on *E. coli* surfaces, clearly showing that compared to T-RF&PMOs nanoparticles, only little amount of J-RF&PMO and RF@PMO nanoparticles is adhered on bacteria.

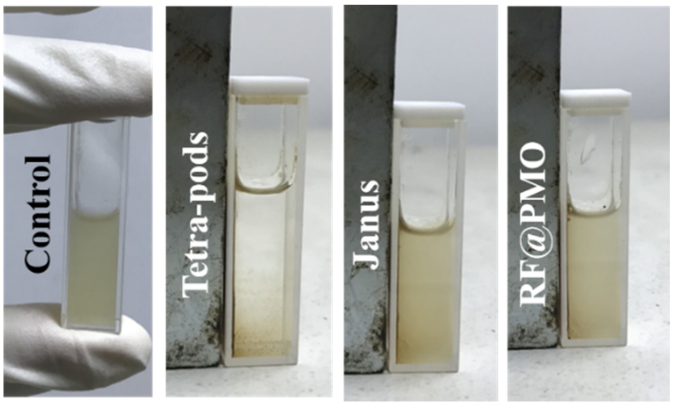

Supplementary Figure 25. Magnetic induced separation of *E. coli*. The digital photos of the PBS solutions after the magnetic induced *E. coli* separation using three kinds of nanoparticles (tetra-pods Fe_3O_4@SiO_2@RF&PMOs nanoparticles, Janus Fe_3O_4@SiO_2@RF&PMO nanoparticles, core@shell Fe_3O_4@SiO_2 @RF@PMO nanoparticles). The nearly transparent culture solution can be obtained after magnetic induced separation of *E. coli* by the tetra-pods Fe_3O_4@SiO_2@RF&PMOs nanoparticles, indicating the high bacterial segregation efficiency. In comparison, the culture solutions obtained from magnetic induced separation of *E. coli* by the other two kinds of nanoparticles are more turbid. Note: the fewer bacteria there are in solution, the clearer the solution is.

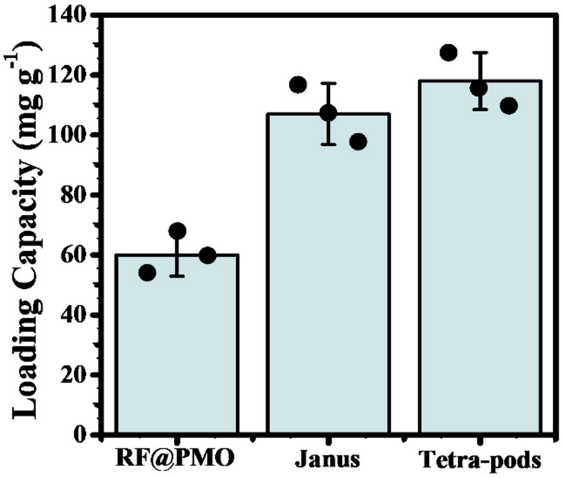

Supplementary Figure 26. The lysozyme loading capacity of various nanoparticles. The lysozyme loading amount for the three kinds of nanoparticles (tetra-pods Fe_3O_4@SiO_2@RF&PMO nanoparticles, Janus Fe_3O_4@SiO_2@RF&PMO nanoparticles, core@shell Fe_3O_4@SiO_2@RF@PMO nanoparticles), showing that the tetra-pods Fe_3O_4@SiO_2@RF&PMO nanoparticles have a relative higher drug loading amount because of the presence of abundant mesopores in the nanoparticles. Source data are provided as a Source Data file.

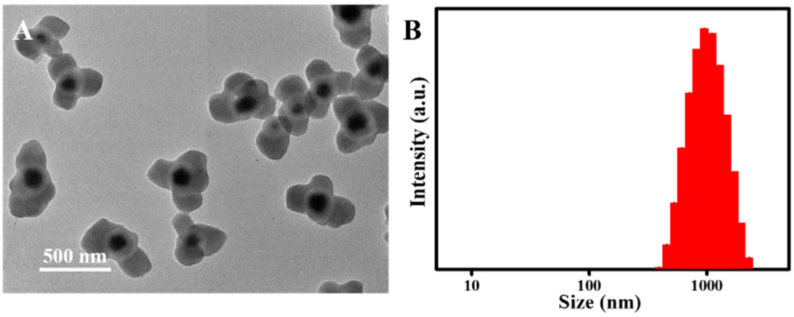

Supplementary Figure 27. The stability test of the nanoparticles after lysozyme loading. (A) TEM images and (B) DLS measurement of lysozyme loaded tetra-pods Fe_3O_4@SiO_2@RF&PMOs nanoparticles. The results confirm that the surface topological structure and good dispersity in Luria-Bertani (LB) media is maintained after lysozyme loading.

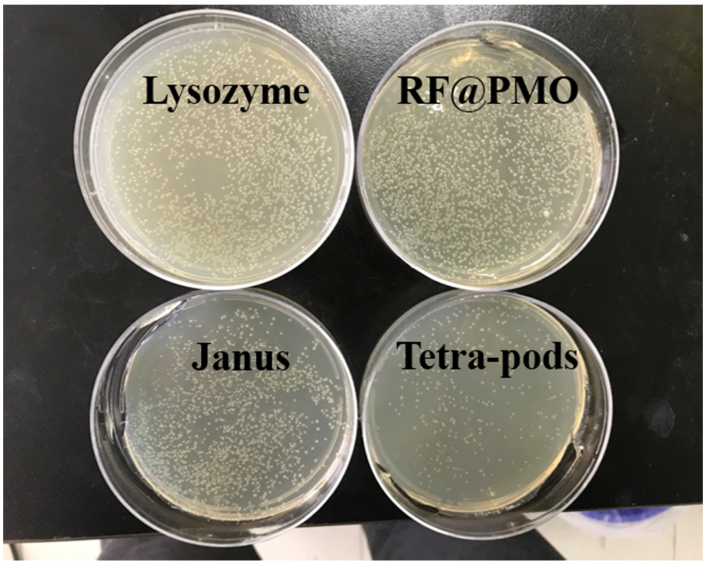

Supplementary Figure 28. The *E. coli* inhibition evaluation of different nanocomposites. The digital photos of the agar plates assessed antibacterial activities after incubating the *E. coli* with three kinds of lysozyme loaded nanoparticles (tetra-pod Fe_3O_4@SiO_2@RF&PMOs nanoparticles, Janus Fe_3O_4@SiO_2@RF&PMO nanoparticles, core@shell Fe_3O_4@SiO_2@RF@PMO nanoparticles) for three days. The result indicates the significant better bacterial suppression ability of tetra-pods Fe_3O_4@SiO_2@RF&PMOs nanoparticles compared to the other two kinds of nanoparticles without multipods topology.

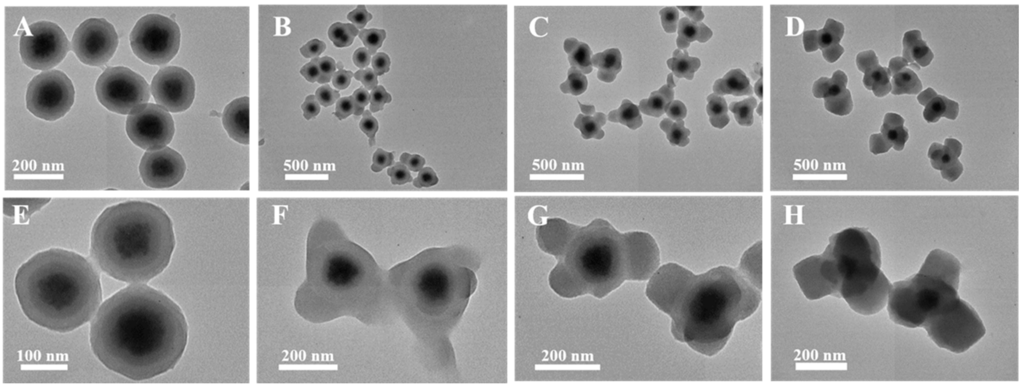

Supplementary Figure 29. Morphologies of nanoparticles obtained at various reaction time. TEM images with different magnifications of tetra-pods Fe_3O_4@SiO_2@RF&PMOs nanoparticles obtained at (A, E) 10 min, (B, F) 20 min, (C, G) 40 min, (D, H) 60 min of the reaction. The multi-site nucleation of PMO pods can be observed at 20 min, indicating that the nucleation of PMOs on RF starts earlier than 20 min.

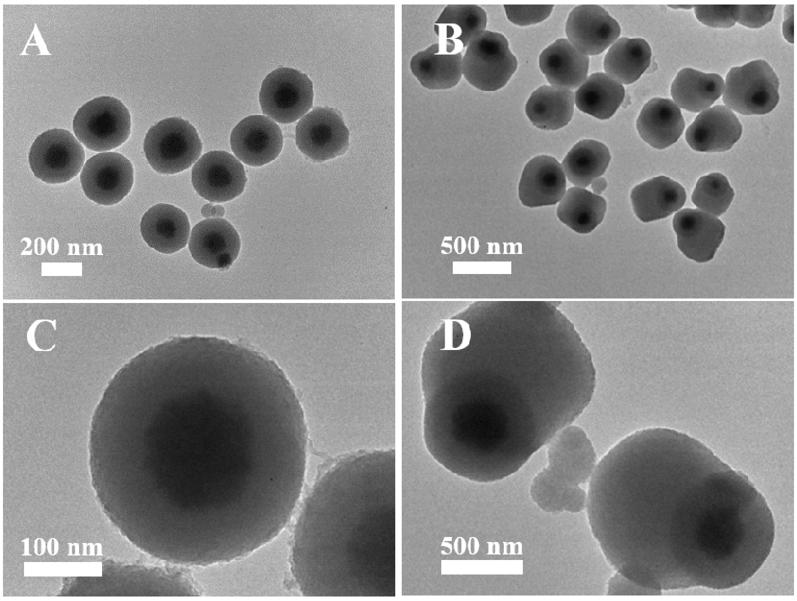

Supplementary Figure 30. Morphologies of nanoparticles obtained at various reaction time. TEM images with different magnifications of Janus Fe_3O_4@SiO_2&PMO nanoparticles obtained at (A, C) 30 min and (B, D) 60 min of the reaction. Comparing to the multipods Fe_3O_4@SiO_2@RF&PMOs nanoparticle (Supplementary Fig. 28), nucleation of PMO on SiO_2 started later.

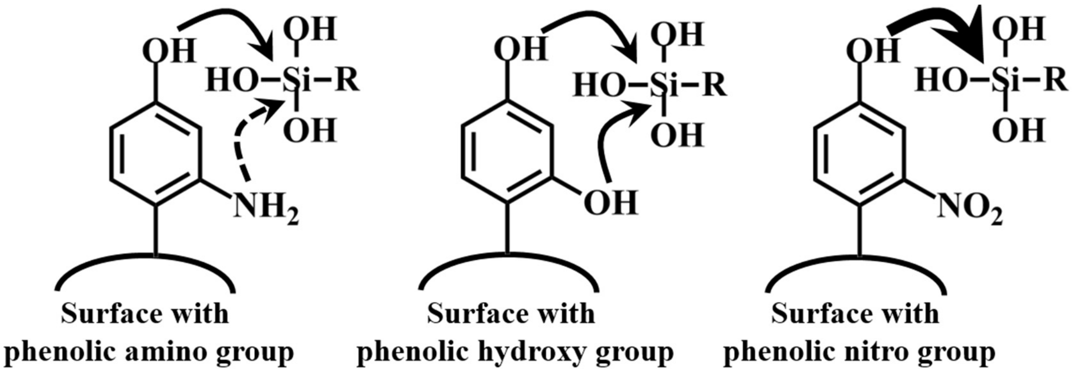

Supplementary Figure 31. Surface reaction kinetics of different nanoparticles. The schematic illustration of the surface reaction kinetic of the silane condensation on the Fe_3O_4@SiO_2@RF-A (phenolic amino group functionalized), Fe_3O_4@SiO_2@RF and Fe_3O_4@SiO_2@RF-N (phenolic nitro groups functionalized) nanoparticles. The surface kinetics of the silane condensation on RF-N is the fastest, and that on RF-A surface is the slowest among the three, thus the nucleation property of PMO would be different on the three kinds of nanoparticles.

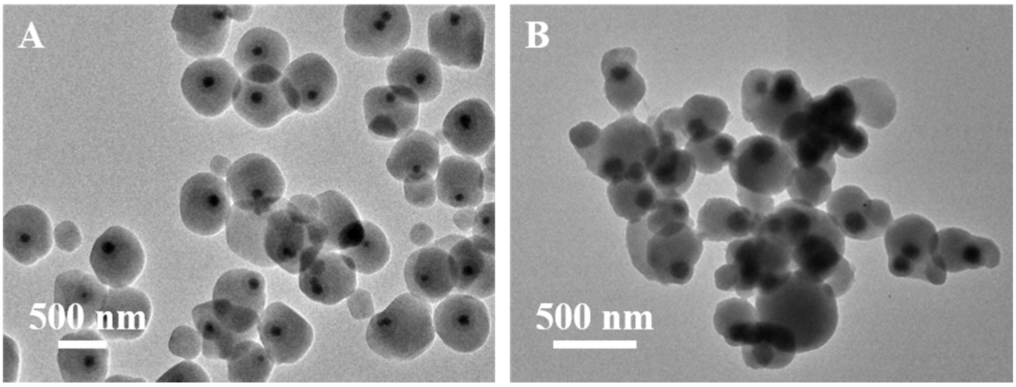

Supplementary Figure 32. The nanoparticles obtained by increasing solution reaction kinetics. TEM images of (A) the Janus Fe_3O_4@SiO_2&PMO nanoparticles by increasing the concentration of the silane precursor (BTEE) from $3.4*10^{-3}$ to $7.0*10^{-3}$ M. (B) the Janus Fe_3O_4@SiO_2&PMO nanoparticles by increasing the concentration of ammonium hydroxide from 0.55 to 1.0 M. It can be seen that the nucleation site number cannot be changed by increasing the concentration of the silane precursors or ammonium hydroxide. The poly-disperse Janus Fe_3O_4@SiO_2&PMO nanoparticles and pure PMO nanoparticles are observed in the samples.

Supplementary Methods

Chemicals:

Hexadecyltrimethylammonium bromide (CTAB, 99 %), tetraethyl orthosilicate (TEOS, AR), ethanol, iron (III) chloride hexahydrate ($FeCl_3 \cdot 6H_2O$, AR), ethylene glycol (AR), sodium citrate tribasic dehydrate (NaAc) (98 %), resorcinol (99 %), 3-aminophenol (99 %), 3-nitrophenol (99 %), formaldehyde solution (37 wt. % in H_2O), Pluronic® F-127, polyvinyl pyrrolidone (PVP, mol weight 40000) were purchased from Sigma-Aldrich. Ammonium hydroxide solution (28 % NH_3 in H_2O), 1,3,5-trimethylbenzene (TMB, AR), sodium hydroxide (NaOH, AR), carbon nanotube (CNT), ethanol were obtained from Shanghai Chemical Reagents Co. Ltd. Bis(triethoxysilyl)ethane (BTEE) and 1,4-bis(triethoxysilyl)benzene (BTEB) were purchased from Gelest Inc. *Escherichia coli* (*E. Coli*, ATCC No. 12435) was selected for bacterial experiments. All chemicals were used as received without any further purification.

Synthesis of core@shell@shell Fe_3O_4@SiO_2@RF nanoparticles:

The preparation process of the core@shell@shell Fe_3O_4@SiO_2@RF nanoparticles was listed in the text of this paper. For the preparation of phenolic amino group or phenolic nitro group functionalized Fe_3O_4@SiO_2@RF nanoparticles, 3-aminophenol or 3-nitrophenol was mixed with resorcinol at a molar ration of 1:1 and used as a precursor for the RF layer coating. Other conditions were retained unchanged.

Synthesis of core@shell Fe_3O_4@RF nanoparticles:

The coating of RF layers on Fe_3O_4 nanoparticles was realized through an extended Stöber method[3]. 40 mg of Fe_3O_4 nanoparticles was dispersed in a mixture of ethanol (96 mL), H_2O (48 mL), resorcinol (325 mg) and formaldehyde (0.325 mL). Then, ammonium hydroxide (25 wt. %, 2.40 mL) was added dropwise and stirred for another 5 h at room temperature. The products were centrifuged and washed with water and ethanol.

For the preparation of other RF coated nanomaterials, for example, the spindle α-Fe_2O_3, carbon nanotubes (CNTs) and graphene nanosheets, the reaction procedure was the same as Fe_3O_4 nanoparticles.

Synthesis of core@shell Fe_3O_4@mRF nanoparticles:

The coating of mesoporous RF layers was carried out through an modified emulsion induce assembly[4]. For the preparation of the core@shell Fe_3O_4@mRF nanoparticles with a diameter of 200 nm, 10 mg of the magnetic Fe_3O_4 nanoparticles above was dispersed in a mixture containing ethanol (10 mL), H_2O (10 mL), Pluronic F127 (0.20 g), TMB (0.40 mL), resorcinol (30 mg) and formaldehyde (30 μL). The mixture was stirred for 30 min to form a uniform emulsion, then ammonium hydroxide (25 wt. %, 80 μL) was added dropwise. The mixture was centrifuged after 2

h and washed with water and ethanol to obtain the products.

For the fabrication of the core@shell structured Fe_3O_4@mRF with a diameter of 300 nm, the addition amounts of resorcinol and formaldehyde were increased to 60 mg and 60 μL, respectively, and reaction time was increased to 12 h.

Synthesis of tetra-pods Fe_3O_4@SiO_2@RF&PMOs nanoparticles (T-RF&PMOs):

The asymmetric growth and assembly of PMO cubes on the Fe_3O_4@SiO_2@RF nanoparticles to form tetra-pods topology was carried out through a surface-kinetics mediated multi-site nucleation strategy, and the detailed method was listed in the text. For the preparation of other kinds of multipods nanocomposites, the Fe_3O_4@SiO_2@RF nanoparticles were replaced with Fe_3O_4@mRF, Fe_2O_3@SiO_2@RF, GR@RF and CNT@RF, respectively.

For the preparation of Janus Fe_3O_4@SiO_2@RF&PMO nanoparticles (J-RF&PMO), the Fe_3O_4@SiO_2@RF nanoparticles obtained above were first dispersed in PVP aqueous solution (5.0 mL, 1.0 mg mL^{-1}), stirred for 30 min, centrifuged and then dispersed in the reaction mixture for PMO growth.

Synthesis of core@shell Fe_3O_4@SiO_2@RF@PMO nanoparticles:

Uniform PMO coating was realized through a modified Stöber method[5]. 10 mg of the Fe_3O_4@SiO_2@RF nanoparticles obtained above were dispersed in ethanol (30 mL), H_2O (7.5 mL), CTAB (80 mg) and ammonium hydroxide (25 wt. %, 1.0 mL). BTEE (50 μL) was then added dropwise. The product was centrifuged after 5 h and washed with water and ethanol.

Synthesis of Fe_3O_4@SiO_2@RF&PMOs nanoparticles with multiple nanorods:

10 mg of Fe_3O_4@SiO_2@RF nanoparticles were centrifuged and dispersed in the mixture of H_2O (38 mL) and ethanol (2.0 mL). Then, CTAB (150 mg) and ammonium hydroxide (25 wt. %, 1.8 mL) were added in succession. The mixture was stirred for 12 h, then BTEB (20 μL) was added dropwise. The products were centrifuged after 3 h and washed with water and ethanol.

Particle stability examination:

Three kinds of nanoparticles are dispersed in different solutions: Luria-Bertani (LB) media, phosphate buffer saline (PBS), acid and basic solutions with a concentration of 0.5 mg mL^{-1}. The particle concentration was the same as the bacterial incubation condition. The dispersion was shaken at 200 rpm and taken at certain time for dynamic light scattering (DLS) measurements.

Bacteria separation tests under external magnetic field:

E. coli was cultured in LB media (tryptone 10 g L^{-1}, yeast extract 5.0 g L^{-1}, NaCl 10 g L^{-1}, pH 7.0) at 37 °C under shaking at 200 rpm for 12 h. Then, bacteria suspension (100 μL, 5×107 CFU mL^{-1}) was diluted with LB medium (800 μL), followed by the addition of PBS solution of T-RF&PMO

nanoparticle (100 μL, 5.0 mg mL^{-1}). The mixtures were further incubated in 37 °C under shaking at 200 rpm for 12 h. Then a magnet was employed to separate the Fe_3O_4 containing T-RF&PMO, J-RF&PMO and RF@PMO nanoparticles. The remaining bacteria number in the solution was determined according to the optical density (OD) of the solution at 600 nm.

Lysozyme loading:

Typically, 1.0 mg of the nanoparticles (T-RF&PMOs, J-RF&PMO and RF@PMO) was dispersed in PBS (1.0 mL) by ultrasonication, and then mixed with lysozyme PBS solution (1.0 mL, 2.0 mg mL^{-1}). After rotating at room temperature for 24 h, the mixtures were centrifuged. The residual lysozyme content was measured by using UV-vis spectrophotometer at a wavelength of 285 nm. The lysozyme loading amounts were calculated based on the original and residual concentrations.

Bacteria inhibition tests:

To investigate the antibacterial activity of lysozyme-free and -loaded nanoparticles, bacterial viability was determined at varied concentration of lysozyme. Typically, bacterial suspension (100 μL of 5×10^7 CFU mL^{-1}) and EDTA (100 μL, 10 mM) were added into the LB medium (700 μL) for each 1.5-mL centrifuge tubes. Then, 200 μL of the samples (lysozyme and lysozyme loaded nanoparticles diluted to certain lysozyme concentration) in PBS was added and shaken at 37 °C on a shaker bed at 200 rpm for 24 h. PBS was added as a control group. The bacterial viability was determined by the optical density (OD) readings at 600 nm (background subtracted) compared with the PBS control group.

Characterization:

Transmission electron microscopy (TEM), high-resolution transmission electron microscopy (HRTEM), high-angle annular dark field imaging in the scanning TEM (HAADF-STEM) observations were acquired on JEM-2100F transmission electron microscope with an accelerating voltage of 200 kV equipped with a post-column Gatan imaging filter (GIF-Tridium). The samples for TEM measurements were suspended in ethanol and supported onto a carbon film on a Cu grid. Scanning electron microscopy (SEM) images were taken using a Hitachi S-4800 ultrahigh resolution cold FEG with an in-lens electron optic operating at 20 kV. Nitrogen adsorption-desorption measurements were conducted to obtain information on the porosity. The measurements were conducted at 77 K with ASAP 2420 and Micromeritcs Tristar 3020 analyzer (USA). Before measurements, the samples were degassed in vacuum at 120 °C for at least 12 h. Small-angle X-ray scattering (SAXS) measurements were taken on a Nanostar U small angle X-ray scattering system (Bruker, Germany) using Cu Kα radiation (40 kV, 35 mA). Magnetic measurements were carried out using a TDM-B vibrating sample magnetometer (VSM) at 300 K. UV/Vis spectra were recorded on Lambda 35 PerkinElmer.

Statistical analysis:

The statistical analysis was performed using one-way analysis of variance (ANOVA), followed by post hoc Tukey's method to test all pair-wise mean comparisons. The results are expressed as mean standard deviation. A value of **P < 0.01 for all the tests was considered of statistical significance.

Supplementary References

1. Liu, J. et al. Highly water-dispersible biocompatible magnetite particles with low cytotoxicity stabilized by citrate groups. *Angew. Chem. Int. Ed.* **48,** 5875-5879 (2009).
2. Deng, Y., Qi, D., Deng, C., Zhang, X. & Zhao, D. Y. Superparamagnetic high-magnetization microspheres with a Fe_3O_4@SiO_2 core and perpendicularly aligned mesoporous SiO_2 shell for removal of microcystins. *J. Am. Chem. Soc.* **130,** 28-29 (2008).
3. Liu, J. et al. Extension of the Stöber method to the preparation of monodisperse resorcinol–formaldehyde resin polymer and carbon spheres. *Angew. Chem. Int. Ed.* **123,** 6069-6073 (2011).
4. Guan, B., Yu, L. & Lou, X. Formation of asymmetric bowl-like mesoporous particles via emulsion-induced interface anisotropic assembly. *J. Am. Chem. Soc.* **138,** 11306-11311 (2016).
5. Li, X. et al. Degradation-restructuring induced anisotropic epitaxial growth for fabrication of asymmetric diblock and triblock mesoporous nanocomposites. *Adv. Mater.* **29,** 1701652 (2017).
